# Effect of perioperative music on delirium after hip fracture operations (MCHOPIN): a multicentre randomised clinical trial in Dutch hospitals

**DOI:** 10.1136/bmjopen-2024-095819

**Published:** 2025-08-28

**Authors:** Thomas LA Dirven, Victor X Fu, Antonia S Becker, Eleni-Rosalina Andrinopoulou, Kees Kalisvaart, Johannes Jeekel, Markus Klimek, Michael HJ Verhofstad, MCHOPIN Collaborators, Detlef van der Velde

**Affiliations:** 1 Department of Neuroscience, Erasmus MC, Rotterdam, The Netherlands; 2 Erasmus MC University Medical Center Rotterdam, Rotterdam, The Netherlands; 3 Spaarne Gasthuis Haarlem-noord, Haarlem, The Netherlands; 4 Anesthesiology, Erasmus MC, University Medical Centre, Rotterdam, The Netherlands; 5 Trauma Surgery, Erasmus MC, University Medical Center Rotterdam, Rotterdam, The Netherlands

**Keywords:** Delirium, Aged, Orthopaedic & trauma surgery

## Abstract

**Objectives:**

Postoperative delirium is a frequent complication with possible detrimental consequences in older hip fracture patients. Music interventions are promising, with positive effects on risk factors for delirium. This study aimed to assess the impact of perioperative music on postoperative delirium in older hip fracture patients.

**Design:**

Prospective randomised controlled trial.

**Setting:**

Multicentre study, performed in six participating hospitals in the Netherlands.

**Participants:**

Eligibility criteria included patients aged ≥65 years with an acute hip fracture requiring surgery and documented informed consent. 449 patients were randomised, with a median age of 81 years (IQR 74–87), including 287 women (63.9%).

**Interventions:**

Music group participants received the intervention preoperatively, intraoperatively, and postoperatively twice a day for 30 min. The control group received standard-of-care, supplemented by headphones without music intraoperatively for equal noise reduction in both groups.

**Primary and secondary outcome measures:**

The primary outcome was delirium diagnosis (Diagnostic and Statistical Manual of Mental Disorders, fifth edition), assessed by a geriatrician. Associations were analysed using regression models. Secondary outcomes included: Delirium Observational Score, anxiety, pain and postoperative complications.

**Results:**

Intention-to-treat analysis showed no statistically significant decrease of delirium in the music group, compared with the control group (OR 0.685 (95% CI 0.378 to 1.242); p=0.21). However, in the modified-intention-to-treat analysis, a significant decrease in postoperative delirium was observed (OR 0.478 (95% CI 0.245 to 0.933); p=0.028), which is substantiated by a logistic regression (OR 0.43 (95 % CI 0.19 to 0.98); p=0.045). Also, more postoperative complications were observed in the control group (93 (43.3%); 66 (32.7); p=0.026) in this analysis. The intervention was associated with high patient satisfaction and no adverse events.

**Conclusions:**

This study suggests a positive effect of music interventions on postoperative delirium, which provides additional evidence for considering the implementation of these interventions in hip fracture care.

**Trial registration number:**

International Clinical Trial Registry Platform, Dutch Trial Register (www.onderzoekmetmensen.nl/, ID:NTR7036).

**STRENGTHS AND LIMITATIONS OF THIS STUDY**

- This multicentre randomised controlled trial was conducted in six hospitals with a substantial sample size.
- The music intervention is safe, non-invasive and not associated with any complications for patients.
- Validated and clinically relevant outcome measures (Diagnostic and Statistical Manual of Mental Disorders, fifth edition and Delirium Observational Score) were used to assess delirium.
- Blinding was not feasible due to the nature of the intervention and the clinical assessment of delirium.

## Introduction

 Hip fractures or proximal femur fractures are a substantial healthcare problem, which occur frequently in aged patients. The number of hip fracture patients was around 75 000 in 2022 in the UK^1^ and 337 438 in the USA in 2018.^2^ In Dutch hospitals, one out of four acute admissions was due to these fractures in 2020.^3^ With the demographic shift in ageing population and increased life expectancy, the incidence is expected to rise to 4.76 million in 2050 worldwide.^4 5^ The majority of these patients are treated surgically, that is, more than 70 %.^6^

Postoperative delirium (POD) is one of the most common complications after acute hip fracture surgery in older individuals, with an incidence ranging 19–37%.^7^,^10^ Delirium is described as an acute disturbance in attention and awareness with additional disturbance in cognition, not better explained by a pre-existing, established or evolving neurocognitive disorder.^11^ Furthermore, it is associated with an increased number of complications, a higher risk of dementia,^12 13^ longer lengths of stay,^14 15^ more readmissions,^16^ higher medical costs^15^ and increased mortality.^12^ Previous studies show that non-pharmacological interventions may have a beneficial effect on delirium in aged patients,^17 18^ without the side effects of pharmacological interventions.

Specifically, music interventions surrounding operations are promising, with proven beneficial effects on patient outcomes, in particular, a decrease in anxiety, pain,^19 20^ stress response,^21^ intraoperative midazolam and postoperative opiate requirements,^22^ all possible factors which may provoke a delirium.^23^,^27^ A recent meta-analysis^28^ found that music interventions may be effective in reducing delirium. However, the included studies showed substantial heterogeneity and the sample sizes were relatively small (n=22–147), particularly in studies focusing on patients undergoing hip surgery. Also, none of the studies used a delirium diagnosis based on Diagnostic and Statistical Manual of Mental Disorders, fifth edition (DSM-5) criteria. This study addresses these gaps by conducting a multicentre randomised clinical trial in older hip fracture patients, using DSM-5 criteria.

The objective of this trial was to investigate the effect of perioperative music on incidence of POD in older patients undergoing hip fracture surgery.

## Methods

### Trial design

**Figure 1:**
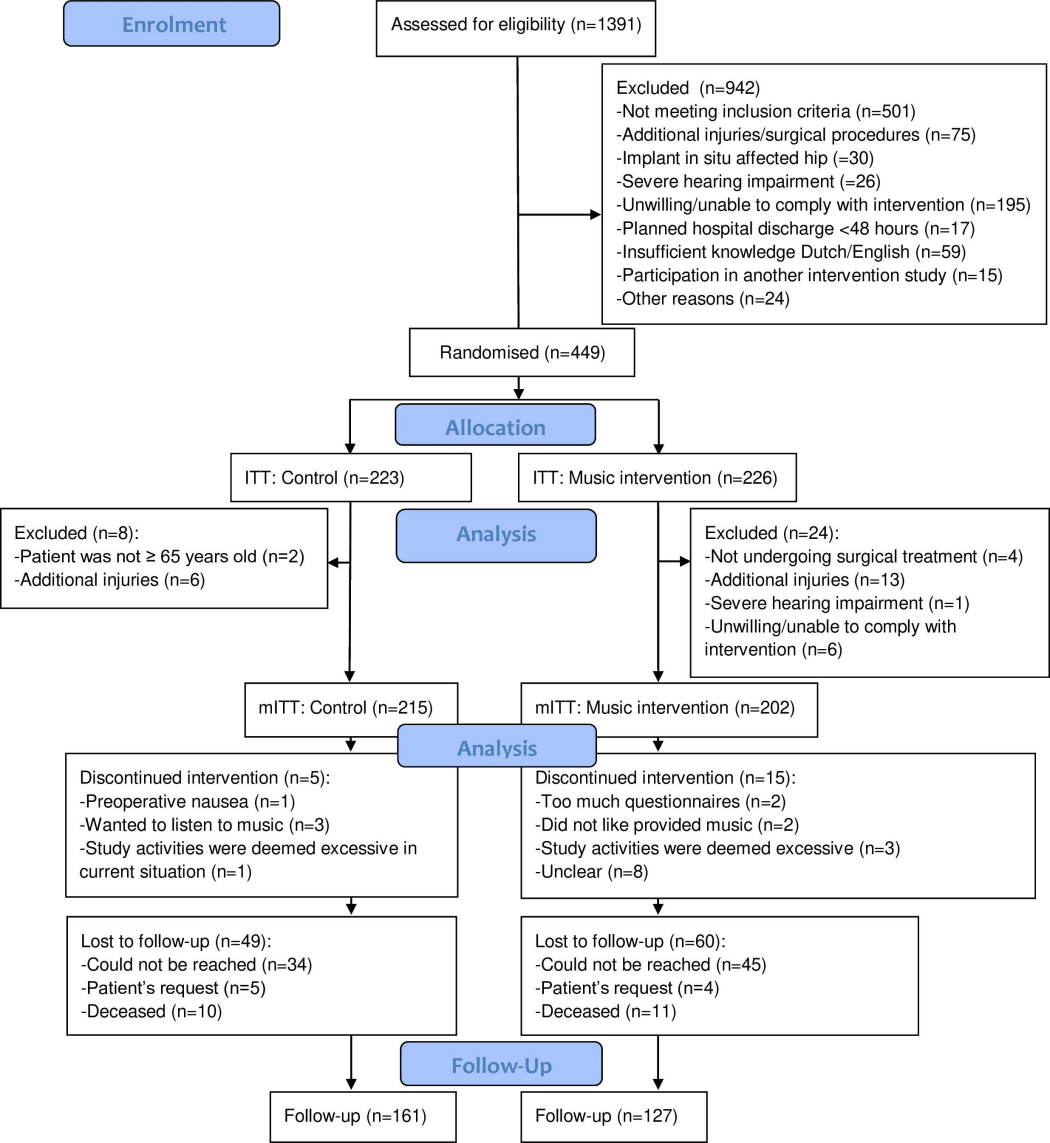
CONSORT 2010 flow diagram. CONSORT, Consolidated Standards of Reporting Trials; ITT, intention-to-treat; mITT, modified intention-to-treat.

This two-arm parallel multicentre randomised controlled trial (RCT) was conducted from March 2019 to August 2023 in six Dutch hospitals (Sint Antonius Hospital, Utrecht; Onze Lieve Vrouwe Gasthuis, Amsterdam; Reinier de Graaf Gasthuis, Delft; Haga Hospital, The Hague; Spaarne Gasthuis, Haarlem; Alrijne Hospital, Leiderdorp). The study was approved by the Medical Ethics Review Board of Erasmus MC (MEC-2018–110, NL64721.078.18) and locally approved in participating hospitals. The research project is performed in accordance with the principles of the Helsinki Declaration. It was registered in the Overview of Medical Research in the Netherlands (www.onderzoekmetmensen.nl/, ID:NTR7036) and therefore registered in International Clinical Trial Registry Platform. Further details of the study are shown in the previously published protocol, including more information on study population, procedures and outcome measures.^29^ This study is reported following the Consolidated Standards of Reporting Trials (CONSORT) 2010 statement^30^ (see figure 1 and online supplemental table S1).

### Study population

Possible participants were eligible for inclusion if they met the following criteria: a proximal femur fracture requiring surgical treatment, age ≥65 years, provision of written informed consent by the patient or a legal proxy. Exclusion criteria were:

- Additional serious injuries or additional surgical procedures that may affect any of the outcome parameters.
- Simultaneous bilateral hip fracture.
- Implant in situ in the affected hip.
- Severe hearing impairment, defined as no verbal communication possible.
- Patients unwilling or unable to comply with the intervention.
- Preoperative planned hospital discharge and return to nursing home within 48 hours of admission.
- Insufficient knowledge of the Dutch or English language to understand the study documents in the judgement of the attending physician or researcher.
- Participation in another intervention study that may influence the duration of surgery or any of the outcome parameters.

Suitable patients were approached preoperatively by a physician in the emergency department or on the clinical ward. Written informed consent was obtained on approval by the patient or their legal representative. Participants were allocated by a secured computerised web-based randomisation programme to either the intervention group or the control group in a 1:1 ratio stratified by site.

### Intervention

Patients in the music group received music for at least 15 min before surgery, during the operation and postoperatively for five consecutive days or until discharge, in addition to standard care. Postoperatively, the intervention was provided daily two times for 30 min, based on the previous literature,^19 22^ in the morning and the evening after the clinical ward rounds. Patients could select music of their choice from preselected playlists (see online supplemental table S2) as well as from a wide variety of music available on a tablet. After the inclusion of 94 music group participants, the platform to provide music was changed from AIMP app to Spotify. It remains unclear whether specific genres are more effective in preventing delirium. However, prior research suggests that personalised music is more effective than fixed genres,^31^ which supports our use of patient-preferred music. A volume limitation was used on the tablets to avoid harmful high volumes. Nurses were instructed to support participants in the music group with the intervention. The control group participants received instructions and support not to listen to music preoperatively, intraoperatively and postoperatively. Subjects in the control group received standard-of-care and no changes in routine care, apart from the headphones intraoperatively without music, in order to differentiate between the effect of music itself and a possible effect of noise, which can have detrimental effects on patient outcome.^32^ Nurses were trained regularly by a local coordinator and periodically, around every 6 months, a presentation was provided to explain the procedures. From January 2022 to the end of the study period, nurses were called twice daily every weekday to support adherence to the protocol. Fidelity of the intervention was assessed by nurses at each intervention moment and was recorded on the form or in the electronic patient file.

### Outcome measures

The primary outcome was presence or absence of POD in the seven postoperative days or till discharge. Continuous and coordinated multidisciplinary orthopaedic-geriatric assessment and review is part of the Dutch Hip Fracture Guidelines^33^ and National Institute for Health and Care Excellence.^34^ First, nurses evaluated patients using the Delirium Observational Screening (DOS) Scale, a validated 13-item tool.^35 36^ The DOS Scale remains the preferred instrument in the Netherlands due to its integration in routine care.^37^ Second, if patients had a mean DOS score of ≥3, a geriatrician was consulted to confirm the diagnosis of POD, based on the DSM-5 criteria.^11^ Moreover, geriatricians were actively involved in hip fracture care of older individuals in all participating centres and they could also assess patients who did not have a previously elevated DOS score. Anaesthesia depth was monitored using Bispectral Index or equivalent devices where available. Despite evidence suggesting music may reduce propofol requirements,^22^ during hip fracture surgery, most centres used volatile agents, and sedative dosages were not systematically recorded.

To further explore the impact of music, secondary outcomes were analysed, as described in the previously published protocol.^29^ Anxiety was measured using the Six-Item State-Trait Anxiety Inventory (STAI-6), a validated short form of the original 20-item STAI-State subscale. The STAI-6 was administered preoperatively in the emergency department or surgical ward and on postoperative days 1 and 2. Cognitive functioning was preoperatively evaluated by a physician, using a three-item questionnaire, the Mini-Cog.^38^ This questionnaire highly correlates to the cognitive function assessment using Mini-Mental State Examination.^39^ As decreased cognitive functioning is associated with an increased risk of POD, this variable was measured preoperatively.^40^ Following the Good Clinical Practice standards, all collected data were stored confidentially in the electronic data capture programme CastorEDC (Ciwit B.V., Amsterdam). Additionally, the predefined cost-effectiveness analysis will be performed and published in another manuscript. In the previously published protocol, it was described that cortisol samples would be assessed from the participants. However, this study procedure was cancelled due to the increased work load, absent qualifications of nurses and hospital logistics in the acute setting.

### Statistical analysis

The POD prevalence in hip fracture patients who were over 65 years old ranged from 19% to 37% in the Netherlands.^8 41^ A reduction of 13%in delirium rates was found in a previous meta-analysis, which mainly investigated non-pharmacological interventions.^42^ Thus, we aimed to find this minimal clinically relevant reduction, taking into account a power of 80% and an alpha of 5% with two-sided testing. We adjusted the sample for possible in-hospital mortality and loss to follow-up of 10%. This resulted in an intended sample size of 452 patients in total (226 patients per group). Initially, we based our sample size on delirium incidence rates reported in international studies and calculated a sample size of 508, as described in the previously published protocol.^29^ However, during the study, a protocol amendment was submitted and approved by the Medical Ethics Review Board of Erasmus MC. The revised sample size calculation, based on Dutch delirium incidence rates, resulted in a target of 452 participants.

The analyses were executed on an intention-to-treat (ITT) basis and a modified-intention-to-treat (mITT) basis. In the analysis on an ITT basis, all registered and randomised patients are investigated, while in the mITT analysis the same population is investigated without patients who were found ineligible after randomisation, using the in- and exclusion criteria after randomisation. In clinical trials involving acutely ill patients requiring urgent interventions—such as individuals undergoing surgery for hip fractures—the assessment of eligibility for inclusion may be challenged by time constraints and clinical urgency. Consequently, ineligible patients may be inadvertently enrolled and randomised due to human error. To address this, a strict mITT analysis was applied after data collection.^43^ For example, patients were excluded from the mITT analysis under the criterion ‘unwilling/unable to comply with intervention’, only if this was explicitly documented in the electronic patient file and if the patient had not received any part of the intervention.

All data were analysed using the Statistical Package for the Social Sciences V.24 or higher. The Shapiro-Wilk test was used to test the normality of the continuous data. When the data were normally distributed, the mean and SD were reported. When data were not normally distributed, the median and percentiles were described. For the testing of variables between groups, the Mann-Whitney U test or Student’s t-test was performed on continuous data and χ^2^ or Fisher’s exact test was performed on categorical data. Analyses were done for outcomes in both ITT and mITT analysis. Adjusted and unadjusted ORs were calculated.

A logistic regression model was developed to correct for possible confounding variables, which were added based on previous literature.^44^ The following variables were entered into the model: age, American Society of Anesthesiologists (ASA)>=3, delirium prior to admission and cognitive impairment according to Mini-Cog. Statistical significance was defined as an alpha <0.05. Due to our clear primary outcome and hypothesis, no multiple testing was done. Therefore, no corrections were implemented for p values. Also, no corrections for missing data were executed.

Subgroup analyses were performed, as specified a priori, with stratification by age (<80 years and ≥80 years). Additionally, participants without a preoperatively diagnosed delirium were investigated in the groups. Secondary outcomes were analysed in the mITT population. Due to the limited amount of full adherence in the music group, no per-protocol analysis was executed.

## Results

A total of 1391 subjects were assessed for eligibility, of which 942 patients were excluded (see figure 1 CONSORT flow diagram). The trial was not formally paused during the COVID-19 crisis. However, the pandemic caused a severe decrease in the inclusion rates from 2 March 2020 to 3 January 2022, due to the prioritisation of care in Dutch hospitals. The informed consent forms of three included patients were lost and could not be retrieved. As a result, the ITT population comprised 449 patients, instead of the initially calculated 452 at the start of the inclusion period.

The baseline characteristics are presented in table 1. The median age of all participants was 81 years (IQR 74–87) and there were no significant baseline differences between the music and control group in ITT and mITT analysis. POD was diagnosed in 50 patients (11%) in the ITT population with no statistical difference between control and music group (OR 0.685 (95% CI 0.378 to 1.242); p=0.21) (see table 2). When applying the eligibility criteria postrandomisation (mITT analysis), we observed a higher incidence of delirium in the control group, compared with the music group (OR 0.478 (95% CI 0.245 to 0.933); p=0.028). This effect persisted after adjusting for confounders using logistic regression (OR 0.43 (95 % CI 0.19 to 0.98); p=0.045) (see online supplemental table S3 for the logistic regression model).

**Table 1:** Baseline characteristics

	ITT population (n=449)		mITT population (n=417)	
	Control (n=223)	Music (n=226)	Control (n=215)	Music (n=202)
Age (years)*	81 (75–87)	80 (73–87)	81 (75–87)	79 (73–85)
Female (%)	143 (64.1)	144 (63.7)	137 (63.7)	128 (63.4)
Body mass index (kg/m^2^)*	23.79 (21.8–26.3)	24.22 (21.7–26.8)	23.6 (21.19–26.22)	23.88 (10.96–26.61)
Comorbidities*	4 (2 - 6)	3 (2 - 5)	4 (2-6)	3 (2-5)
ASA-classification:†	2 (2-3)	3 (2-3)	2 (2-3)	3 (2-3)
Delirium prior to admission	21 (9.4)	21 (9.3)	20 (9.3)	14 (6.9)
Dementia prior to admission	37 (16.6)	30 (13.3)	36 (16.7)	25 (12.4)
Depression prior to admission	16 (7.2)	15 (6.6)	16 (7.4)	15 (7.4)
Intoxications				
Smoking	41 (18.4)	40 (17.7)	39 (18.1)	38 (18.8)
Alcohol	19 (8.5)	22 (9.7)	19 (8.8)	20 (9.9)
Drugs	2 (0.9)	1 (0.4)	2 (0.9)	1 (0.5)
Number of medications prior to admission*	5 (2-8)	5 (2–7.3)	5 (2-8)	5 (2-7)
Preoperative cognitive impairment‡	69 (34.3)	71 (36.4)	67 (34.7)	59 (33.7)
Preoperative DOS ≥3	16 (10.4)	14 (9.0)	16 (10.7)	12 (8.6)
Preoperative delirium by DSM-5	5 (2.2)	8 (3.5)	5 (2.3)	5 (2.5)
Preoperative haloperidol administration	12 (5.4)	8 (3.5)	0 (0–0)	0 (0–0)
Preoperative opiate use	84 (37.7)	76 (33.6)	82 (38.1)	69 (34.2)
Preoperative pain §	4 (2-6)	4 (3-5)	4 (2-6)	4 (3-5)
Preoperative anxiety ¶	40 (30.83–46.67)	40 (30–47.50)	40 (33.33–50.00)	36.67 (30.0–46.67)
Preoperative pneumonia	2 (0.9)	1 (0.4)	2 (0.9)	1 (0.5)
Preoperative urinary tract infection	14 (6.3)	23 (10.2)	14 (6.5)	20 (9.9)
Anaesthesia type:				
Inhalational	26 (11.7)	25 (11.1)	24 (11.2)	25 (12.4)
Intravenous	91 (40.8)	95 (42.0)	86 (40)	85 (42.1)
Spinal	114 (51.1)	114 (50.4)	112 (52.1)	104 (51.5)
Peripheral nerve block	108 (48.4)	110 (48.7)	105 (48.8)	95 (47.0)
Type of operation:				
Total arthroplasty	8 (3.6)	13 (5.8)	8 (3.7)	12 (6.0)
Hemiarthroplasty	87 (39.0)	88 (38.9)	84 (39.1)	80 (39.8)
Dynamic hip screw	21 (9.4)	24 (10.6)	20 (9.3)	23 (11.4)
Cannulated screws	6 (2.7)	2 (0.9)	6 (2.8)	2 (1.0)
Intramedullary nail	101 (45.3)	93 (41.2)	97 (45.1)	84 (41.8)
Music importance**	7 (6-8)	8 (6-8)	7.5 (6-8)	8 (6-8)
Frequency listening:				
The whole day	47 (21.1)	49 (21.7)	43 (20.0)	45 (22.3)
Some hours per day	72 (32.3)	81 (35.8)	69 (32.1)	75 (37.1)
Some hours per week	28 (12.6)	27 (11.9)	28 (13.0)	24 (11.9)
Never	26 (11.7)	27 (11.9)	25 (11.6)	24 (11.9)
Unknown	50 (22.4)	42 (18.6)	50 (23.3)	31 (16.8)
Played an instrument or sang previously	78 (35)	95 (42)	75 (55.8)	91 (53.5)

Values are n (%), unless otherwise indicated.

* Data are reported in median (IQR).

† Classification of ASA, to assess patient’s pre-anaesthesia medical comorbidities in four classes.

‡ Measured by Mini-COG.

§ Measured by Numerical Rating Scale (ranging 0–10).

¶ Measured by State-Trait Anxiety Inventory 6 (ranging 20–80).

** Measured by an ordinal scale, ranging 0–10, in which 0 is least important and 10 is most important.

ASA, American Society of Anesthesiologists; DOS, Delirium Observational Score; DSM-5, Diagnostic and Statistical Manual of Mental Disorders, fifth edition; ITT, intention-to-treat; mITT, modified intention-to-treat.

**Table 2:** Postoperative delirium: univariate and multivariable ORs

	Control	Music	Univariate OR (95% CI’s)	P value*	Multivariable OR (95% CIs)†	P value
Intention-to-treat population	29 (13.0)	21 (9.3)	0.685 (0.378 to 1.242)	0.211	0.656 (0.316 to 1.359)	0.256
Modified Intention-to-treat population‡	29 (13.5)	14 (6.9)	0.478 (0.245 to 0.933)	0.028	0.433 (0.190 to 0.983)	0.045

Values are n (%), unless otherwise indicated.

* Calculated using χ2 test.

† Logistic regression, with following variables entered into the model: age, ASA≥3, delirium prior to admission, cognitive impairment according to Mini-Cog.

‡ Postoperative delirium diagnosed by geriatrician using Diagnostic and Statistical Manual of Mental Disorders, fifth edition.

ASA, American Society of Anesthesiologists.

Subgroup analyses revealed that participants in the music group experienced less POD compared with those in the control group within the mITT population aged 80 years or older (OR 0.377 (95% CI 0.168 to 0.847); p=0.015). This effect was also evident when using logistic regression (OR 0.36 (95% CI 0.14 to 0.92); p=0.034), as shown in table 3. Similarly, in the ITT population aged 80 years or older, excluding patients with preoperative delirium, the music group exhibited a lower incidence of POD with respect to the control group (OR 0.454 (0.211 to 0.976); p=0.04). The initial postoperative day of delirium is presented in online supplemental table S4.

**Table 3:** Subgroup analyses of postoperative delirium: univariate and multivariable ORs

	Control	Music	Univariate OR (95% CI’s)	P value	Multivariable OR (95% CI’s)*	P value
< 80 years old (n=202)						
Intention-to-treat population (ITT)†	3 (3.2)	6 (5.6)	1.784 (0.434 to 7.341)	0.508‡	0.943 (0.177 to 5.013)	0.945
ITT without preoperative delirium†	3 (3.2)	4 (3.8)	1.19 (0.259 to 5.457)	1‡	0.741 (0.132 to 4.159)	0.733
Modified ITT population (mITT)†	3 (3.3)	5 (4.9)	1.497 (0.348 to 6.444)	0.725‡	0.763 (0.136 to 4.278)	0.758
≥ 80 years old (n=247)						
ITT population†	26 (20.2)	15 (12.7)	0.577 (0.289 to 1.152)	0.116§	0.561 (0.245 to 1.285)	0.172
ITT without preoperative delirium†	24 (19.4)	11 (9.8)	0.454 (0.211 to 0.976)	0.04§	0.437 (0.177 to 1.080)	0.073
mITT population†	26 (21.0)	9 (9.1)	0.377 (0.168 to 0.847)	0.015§	0.355 (0.136 to 0.923)	0.034
All ages, without preoperative delirium (n=436)						
ITT population†	27 (12.4)	15 (6.9)	0.523 (0.270 to 1.013)	0.051§	0.534 (0.245 to 1.163)	0.114
mITT population†	27 (12.9)	10 (5.1)	0.362 (0.171 to 0.770)	0.006§	0.355 (0.147 to 0859)	0.022

Values are n (%), unless otherwise indicated.

* Variables entered into the model: age, ASA≥3, delirium prior to admission, cognitive impairment according to Mini-Cog.

† Postoperative delirium diagnosed by geriatrician using Diagnostic and Statistical Manual of Mental Disorders, fifth edition.

‡ Calculated using Fisher’s Exact Test.

§ Calculated using χ2 test.

ASA, American Society of Anesthesiologists.

The secondary outcomes were analysed in the mITT population and are presented in table 4. Patients who received the music intervention had in total fewer postoperative complications compared with the control participants (93 (43.3%); 66 (32.7); p=0.026). No adverse events were found related to the music interventions. The fidelity of the music intervention is shown in online supplemental figure S5.

**Table 4:** Secondary outcomes

	N*	Control	N*	Music	P value
Elevated DOS-score†	215	40 (18.6)	202	31 (15.3)	0.376
Anxiety:‡					
POD-1	93	36.67 (30–43.33)	84	33.33 (30–43.33)	0.62
POD-2	72	36.67 (26.67–43.33)	69	33.33 (26.67–41.67)	0.263
Postoperative pain§	212	2 (2-4)	191	2 (2-3)	0.374
Postoperative medication use:					
Haloperidol (mg)	215	0 (0–0)	202	0 (0–0)	0.242
Opioid use (OME)¶	215	37.50 (15–97.50)	202	37.50 (7.5–76.25)	0.174
Postoperative complications†	215	93 (43.3)	202	66 (32.7)	0.026
Re-interventions†	215	4 (1.9)	202	4 (2.0)	1
Hospital length of stay (days):					
Till medically ready**	107	5 (4-7)	103	5 (4-7)	0.928
Full length of stay	215	7 (5-9)	200	7 (5-9)	0.675
30-day mortality†	211	8 (3.8)	192	9 (4.7)	0.805
90-day readmission†	215	12 (5.6)	202	14 (6.9)	0.569
Functional ability score††	161	6 (3.5–6)	127	6 (4-6)	0.402

All data are presented from the modified intention-to-treat population. Values are median (IQR), unless otherwise indicated. P values were calculated using χ2 test or Fisher’s exact test when appropriate for categorical data and using Mann-Whitney U test for continuous data.

* N represents the number of patients for whom data were available per group.

† Data are presented in number(%).

‡ Measured using State Trait Anxiety Inventory 6, on postoperative day (POD) 1 and 2, respectively.

§ Measured by Numerical Rating Scale (ranging from 0 to 10).

¶ Opioid use was converted in oral morphine equivalents (OME).

** Defined as days from admission date till moment of medically ready, described in electronic medical file by physician.

†† Measured by Katz-ADL (Activities of Daily Living)

DOS, Delirium Observational Score.

## Discussion

We found no significant effect of perioperative music on POD in the ITT analysis. However, when performing the mITT analysis, a positive effect on delirium and postoperative complications was observed (OR 0.478; 95% CI 0.245 to 0.933). The effect on delirium was still present after correction for possible confounders in the mITT analysis.

A recent meta-analysis found a similar preventive effect of music on POD, consistent with our mITT analysis.^28^ When looking at RCTs, which specifically included orthopaedic or trauma surgery patients, four out of five studies found a significant decrease in delirium.^45^,^49^ The remaining study did not find any delirium, possibly due to its low sample size (n=40).^49^ Recently, the effect of music on POD was investigated in a larger sample of neurosurgical patients by Kappen *et al*.^50^ These authors found a significant reduction in DOS scores (OR 0.46, 95% CI 0.19 to 1.00, p=0.048), while no significant reduction was found in DSM-V diagnoses by psychiatrists (OR 0.55, 95% CI 0.14 to 1.96, p=0.34). Delirium is frequently misdiagnosed or unidentified due to the fluctuation of its symptoms, and the psychiatrists in this study may have missed the diagnosis due to their delayed assessment after an elevated DOS.^18^ Moreover, the majority of delirious patients have the hypoactive psychomotor subtype, which is difficult to identify.^51^

In our study, we missed delirium screening and diagnosing as well (see online supplemental table S6). Delirium is usually diagnosed within the first three postoperative days.^52^ In our trial, DOS was not performed in 16.5–34.8% from postoperative day 1–3, despite efforts to stimulate frequent assessments. Our sensitivity analysis in the mITT, including patients with>60% of DOS assessments from postoperative days 1–3, showed an incidence of POD of 14.9% in the control group and 9.6% in the music intervention group. Therefore, the results should be interpreted with caution due to potential under-detection of delirium. Furthermore, our assessed incidence was lower compared with the studies used for our sample size calculation (11.1% vs 19–37%).^7 8 41 53^ We therefore did not detect the previously calculated absolute difference of 13% in the incidence between both groups. However, we did observe a relative reduction in delirium of nearly 49% in mITT analysis (control group 13.5% vs music group 6.9%). In accordance with Dutch guidelines, we used DSM-5 for diagnosing. However, a recent systematic review showed that 3D-CAM (Confusion Assessment Method) offers strong diagnostic accuracy and is less time-consuming. Therefore, we recommend its use in future research to streamline delirium assessment.^54^ We believe that we included a representative sample of hip fracture patients. To enhance generalisability, we did not apply ASA-based exclusion criteria, while a higher ASA classification and prior cognitive impairment are a potential confounder. We adjusted for these confounders in logistic regression and we believe that prior cognitive and psychiatric conditions were evenly distributed due to our randomisation procedure and large sample size. The incidence of delirium prior to admission was assessed using clinical notes. 42 participants (9.4%) had a delirium prior to admission. Delirium in outpatients is also frequently under-recognised, as the symptoms can overlap with symptoms of other diseases. Addessi *et al* found that 13% had delirium of older outpatients with dementia.^55^ Patients with dementia have an increased risk of developing delirium. Therefore, we believe that our measured delirium prior to admission is comparable to other studies (online supplemental table S6).

Adherence to the music intervention was lower compared with similar studies, partly due to the delayed start of adherence tracking and the frailty of the participants (35% were cognitively impaired).^50 56^ Additionally, we believe that limited time among nursing staff contributed to missing data, such as the STAI-6, from the questionnaires.^57^ Conducting research in frail patients in acute care is challenging, but essential to ensure that study findings are applicable to the target clinical population.^58^ Especially considering that a substantial amount of delirium is preventable and non-pharmacological interventions are recommended.^27 59 60^

As for most surgical RCTs and non-pharmacological intervention trials, this study lacks full randomisation blinding,^61 62^ due to the type of intervention. Due to hospital logistics, patients could not consistently be accommodated in single rooms, potentially introducing variability in environmental factors such as ambient noise. Given the complex aetiology and the multitude of factors that may contribute to POD,^44^ we did not adjust for all potential confounding variables, such as intraoperative hypotension^63^ and environmental factors. However, we believe that, given our large sample size and standardised randomisation procedure, confounding factors are evenly distributed across both groups. This is the first multicentre RCT, which investigated the effect of music on POD in older hip fracture patients. The multicentre design and use of validated tools substantiate the generalisability of the results.

The difference in outcomes between our ITT and mITT can be explained by 32 erroneous inclusions in the acute preoperative setting at the emergency department. Of these patients, 19 patients had an additional injury, of which 13 were in the music group. An additional trauma, its accessory inflammation and surgical procedures can increase the risk of developing delirium.^64 65^ Therefore, this may have resulted in relatively more POD in the ITT music group. Despite this possible explanation, the exclusion of randomised patients from the ITT analysis might have resulted in a bias.

Music was associated with less postoperative complications in our mITT analysis, which is in line with a previous systematic review.^12^ Despite our hypothesis and contrary to previous studies,^19^ no reduction in pain or anxiety was observed. Music might not mitigate delirium by modulating its known risk factors. Although the pathophysiology of (postoperative) delirium remains incompletely understood, prior research implicates mechanisms such as neuroinflammation and neurotransmitter imbalance.^18^ Particularly in patients with a hip fracture, pain and surgical stress may induce a systemic release of pro-inflammatory cytokines,^18^ which can influence the hippocampus and amygdala and activate the sympathetic nervous system.^66^ This, in turn, activates the hypothalamic-pituitary-adrenal axis, resulting in elevated cortisol levels, which have been linked to the development of delirium.^67^ Music has been hypothesised to induce anti-inflammatory effects by stimulating the parasympathetic nervous system via the vagal nerve, potentially modulating relevant physiological pathways.^50^ Moreover, music has been associated with reduced serum cortisol levels, which may represent an additional mechanism through which it helps prevent delirium.^21^ Therefore, we encourage future studies to focus on the mechanism of action of music intervention on delirium to elucidate the preventive effect.

The implementation of music interventions in this population should be considered, as the benefits for the patients seem to be worth the effort. We found a high patient satisfaction and no adverse events related to the intervention, which is in line with previous trials.^20 50 68^ 73% of our investigated music group patients would like to listen to music again, in the case that they would be in the same situation. Additionally, the extra workload for nurses, with regard to questionnaires and data collection in addition to the music intervention, is not needed for implementation. This may support the adherence and the effect of the intervention, given the positive outcomes in our mITT analysis. We recommend investigating this effect in a large multicentre RCT, with a focus on standardised validated tools for screening and diagnosing delirium. Additionally, we advocate for a feasible number of study procedures for this frail population.

## Conclusion

This multicentre RCT suggests a beneficial effect of perioperative music interventions on POD in older hip fracture patients. Given that music interventions are relatively inexpensive, with high patient satisfaction and without adverse events, our results add additional evidence to the existing guidelines,^69^ which recommend considering perioperative music interventions.

## Supplementary material

10.1136/bmjopen-2024-095819 online supplemental file 1

## Data Availability

Data are available upon reasonable request.
