# Supplementary material for: Effect of perioperative music on delirium after hip fracture operations (MCHOPIN): a multicentre randomised clinical trial in Dutch hospitals
Source: BMJ Open. 2025 Aug 28;15(8):e095819. doi: 10.1136/bmjopen-2024-095819 (PMC12406818; doi:10.1136/bmjopen-2024-095819)
Supplement: online supplemental file 1 [file bmjopen-15-8-s001.docx]

**Supplementary Data**

**Supplementary table S1. Consort 2010 checklist with 2022 extension**

| Section/Topic | Item No | Checklist item | Page No |
| --- | --- | --- | --- |
| Title and abstract | | | |
|  | 1a | Identification as a randomised trial in the title | 1 |
|  | 1b | Structured summary of trial design, methods, results, and conclusions (for specific guidance see CONSORT for abstracts) | 4,5 |
| Introduction | | | |
| Background and objectives | 2a | Scientific background and explanation of rationale | 6,7 |
|  | 2b | Specific objectives or hypotheses | 6,7 |
| Methods | | | |
| Trial design | 3a | Description of trial design (such as parallel, factorial) including allocation ratio | 7 |
|  | 3b | Important changes to methods after trial commencement (such as eligibility criteria), with reasons | 7-12 |
| Participants | 4a | Eligibility criteria for participants | 8 |
|  | 4b | Settings and locations where the data were collected | 7-12 |
| Interventions | 5 | The interventions for each group with sufficient details to allow replication, including how and when they were actually administered | 8,9 & P* |
| Outcomes | 6a.1 | Provide a rationale for the selection of the domain for the trial’s  primary outcome | 9-11, P* |
|  | 6a.2 | Describe the specific measurement variable (eg, systolic blood  pressure), analysis metric (eg, change from baseline, final value,  time to event), method of aggregation (eg, mean, proportion), and  the time point for each outcome | 9-11 |
|  | 6a.3 | If the analysis metric for the primary outcome represents within subject change, define and justify the minimal important change in  individuals | NA |
|  | 6a.4 | If the outcome data were continuous, but were analysed as  categorical (method of aggregation), specify the cutoff values  used | NA |
|  | 6a.5 | If outcome assessments were performed at several time points  after randomization, state the time points used for the analysis | 9-11 |
|  | 6a.6 | If a composite outcome was used, define all individual  components of the composite outcome | NA |
|  | 6a.7 | Identify any outcomes that were not prespecified in a trial registry  or protocol | NA |
|  | 6a.8 | Provide a description of the study instruments used to assess the  outcome (eg, questionnaires, laboratory tests) along with  reliability, validity, and responsiveness in a population similar to  the study sample | 9-11, P* |
|  | 6a.9 | Describe who assessed the outcome (eg, nurse, parent), and any  qualifications or trial-specific training necessary to administer the  study instruments to assess the outcome | 9-11, P* |
|  | 6a.10 | Describe any processes used to promote outcome data quality  during data collection (eg, duplicate measurements) and after  data collection (eg, range checks of outcome data values), or  state where details can be found | 9-11, P* |
| Sample size | 7a | How sample size was determined | 10-12 |
|  | 7a.1 | Define and justify the target difference between treatment groups  (eg, the minimal important difference) | 10-12 |
|  | 7b | When applicable, explanation of any interim analyses and stopping guidelines | NA |
| Randomisation |  |  |  |
| Sequencegeneration | 8a | Method used to generate the random allocation sequence | 8 |
|  | 8b | Type of randomisation; details of any restriction (such as blocking and block size) | 8 |
| Allocation concealment mechanism | 9 | Mechanism used to implement the random allocation sequence (such as sequentially numbered containers), describing any steps taken to conceal the sequence until interventions were assigned | 8 |
| Implementation | 10 | Who generated the random allocation sequence, who enrolled participants, and who assigned participants to interventions | 8 |
| Blinding | 11a | If done, who was blinded after assignment to interventions (for example, participants, care providers, those assessing outcomes) and how | 18-21 |
|  | 11b | If relevant, description of the similarity of interventions | 8,9 & P* |
| Statistical methods | 12a.1 | Statistical methods used to compare groups for primary and secondary outcomes | 11, 12 |
|  | 12a.2 | Describe any methods used to account for multiplicity in the analysis or interpretation of the primary and secondary outcomes (eg, coprimary outcomes, same outcome assessed at multiple time points, or subgroup analyses of an outcome) | NA |
|  | 12a.3 | Describe methods to assess patterns of missingness (eg, missing  not at random), and describe the methods to handle missing  outcome items or entire assessments | 12 |
|  | 12a.4 | Provide definition of outcome analysis population relating to  protocol nonadherence (eg, as a randomized analysis) | 11, 12 |
|  | 12b | Methods for additional analyses, such as subgroup analyses and adjusted analyses | 11, 12 |
| Results | | | |
| Participant flow (a diagram is strongly recommended) | 13a | For each group, the numbers of participants who were randomly assigned, received intended treatment, and were analysed for the primary outcome | Figure 1 |
|  | 13b | For each group, losses and exclusions after randomisation, together with reasons | Figure 1 |
| Recruitment | 14a | Dates defining the periods of recruitment and follow-up | 7 |
|  | 14b | Why the trial ended or was stopped | NA |
| Baseline data | 15 | A table showing baseline demographic and clinical characteristics for each group | Table 1 |
| Numbers analysed | 16 | For each group, number of participants (denominator) included in each analysis and whether the analysis was by original assigned groups | Table 1-4 |
| Outcomes and estimation | 17a | For each primary and secondary outcome, results for each group, and the estimated effect size and its precision (such as 95% confidence interval) | Table 1-4 |
|  | 17a.1 | Include results for all prespecified outcome analyses or state  where results can be found if not in this report | 13-17, P* |
|  | 17b | For binary outcomes, presentation of both absolute and relative effect sizes is recommended | Table 1-4 |
| Ancillary analyses | 18 | Results of any other analyses performed, including subgroup analyses and adjusted analyses, distinguishing pre-specified from exploratory | 13-17 |
|  | 18.1 | If there were any analyses that were not prespecified, explain why  they were performed | 13-17 |
| Harms | 19 | All important harms or unintended effects in each group (for specific guidance see CONSORT for harms) | 13-17 |
| Discussion | | | |
| Limitations | 20 | Trial limitations, addressing sources of potential bias, imprecision, and, if relevant, multiplicity of analyses | 18-21 |
| Generalisability | 21 | Generalisability (external validity, applicability) of the trial findings | 18-21 |
| Interpretation | 22 | Interpretation consistent with results, balancing benefits and harms, and considering other relevant evidence | 18-21 |
| Other information | | |  |
| Registration | 23 | Registration number and name of trial registry | 7 |
| Protocol | 24 | Where the full trial protocol can be accessed, if available | 7 |
| Funding | 25 | Sources of funding and other support (such as supply of drugs), role of funders | 2, 3  3 |

*P: Further details are found in the previously published study protocol, Fu VX, Jeekel J, Van Lieshout EMM, Van der Velde D, Slegers LJP, Haverlag R, et al. Effect of music on clinical outcome after hip fracture operations (MCHOPIN): study protocol of a multicentre randomised controlled trial. BMJ Open. 2021;11(12):e049706.

**NA: Not Applicable

Citation: Butcher NJ, Monsour A, Mew EJ, et al. Guidelines for Reporting Outcomes in Trial Reports: The CONSORT-Outcomes 2022 Extension. JAMA. 2022;328(22):2252–2264. doi:10.1001/jama.2022.21022

**Supplementary table S2. Preselected Music Playlists**

60’s-80’s

| I'm Not In Love | 10cc |
| --- | --- |
| The Winner Takes It All | ABBA |
| The Free Electric Band | Albert Hammond |
| Can't Get Enough Of Your Love, Babe | Barry White |
| Just The Way You Are | Barry White |
| God Only Knows - Remastered 1996 | The Beach Boys |
| Good Vibrations | The Beach Boys |
| I Get Around | The Beach Boys |
| Just the Two of Us (feat. Bill Withers) | Grover Washington, Jr. |
| Lovely Day | Bill Withers |
| Thanks for Saving My Life | Billy Paul |
| Denis | Blondie |
| Heart Of Glass | Blondie |
| Sunny | Bobby Hebb |
| More Than a Feeling | Boston |
| Glory Days | Bruce Springsteen |
| Streets of Philadelphia | Bruce Springsteen |
| The River | Bruce Springsteen |
| Love Is The Drug | Bryan Ferry |
| Morning Has Broken | Yusuf / Cat Stevens |
| Take Five | The Dave Brubeck Quartet |
| Brothers In Arms | Dire Straits |
| Sultans Of Swing | Dire Straits |
| Jolene | Dolly Parton |
| American Pie | Don McLean |
| Listen to the Music | The Doobie Brothers |
| Always On My Mind | Elvis Presley |
| Blue Moon | Elvis Presley |
| Heartbreak Hotel | Elvis Presley |
| In the Ghetto | Elvis Presley |
| Love Me Tender | Elvis Presley |
| Suspicious Minds | Elvis Presley |
| You're The First, The Last, My Everything - Single Version | Barry White |
| Don't Stop - 2004 Remaster | Fleetwood Mac |
| Dreams - 2004 Remaster | Fleetwood Mac |
| Come Fly With Me | Frank Sinatra |
| I've Got You Under My Skin | Frank Sinatra |
| My Way | Frank Sinatra |
| Strangers In The Night | Frank Sinatra |
| Baker Street | Gerry Rafferty |
| Back Home | Golden Earring |
| Radar Love | Golden Earring |
| Weekend Love | Golden Earring |
| Fire and Rain | James Taylor |
| Something in the Way She Moves | James Taylor |
| You've Got a Friend | James Taylor |
| Hallelujah | Jeff Buckley |
| Hey Joe | Jimi Hendrix |
| Casey's Last Ride | John Denver |
| Rocky Mountain High | John Denver |
| Take Me Home, Country Roads | John Denver |
| This Old Guitar | John Denver |
| Imagine - Remastered 2010 | John Lennon |
| You'll Never Walk Alone | Lee Towers |
| Walk On the Wild Side | Lou Reed |
| What A Wonderful World | Louis Armstrong |
| California Dreamin' | The Mamas & The Papas |
| I Heard It Through The Grapevine | Marvin Gaye |
| What's Going On | Marvin Gaye |
| Beautiful Noise | Neil Diamond |
| Heart Of Gold | Neil Young |
| Don't Let Me Be Misunderstood | Nina Simone |
| My Baby Just Cares For Me | Nina Simone |
| (Sittin' On) the Dock of the Bay | Otis Redding |
| A Whiter Shade of Pale - Original Single Version | Procol Harum |
| Bohemian Rhapsody | Queen |
| I Want To Break Free | Queen |
| Under Pressure | Queen |
| Streets of London | Ralph McTell |
| Unchained Melody | The Righteous Brothers |
| Under My Thumb | The Rolling Stones |
| All I Have to Do Is Dream | Roy Orbison |
| In Dreams | Roy Orbison |
| Oh, Pretty Woman | Roy Orbison |
| You Got It | Roy Orbison |
| Wonderful World | Sam Cooke |
| Samba Pa Ti | Santana |
| San Francisco (Be Sure to Wear Flowers in Your Hair) - Single Version | Scott McKenzie |
| Bridge Over Troubled Water | Simon & Garfunkel |
| El Condor Pasa (If I Could) | Simon & Garfunkel |
| Homeward Bound | Simon & Garfunkel |
| The Sound of Silence | Simon & Garfunkel |
| Little Man | Sonny & Cher |
| Everytime I Think of You - 2000 Remaster | The Babys |
| Here Comes The Sun - Remastered 2009 | The Beatles |
| Lady Madonna - Remastered 2015 | The Beatles |
| Penny Lane - Remastered 2015 | The Beatles |
| We Can Work It Out - Remastered 2015 | The Beatles |
| With A Little Help From My Friends - Remastered 2009 | The Beatles |
| Yesterday - Remastered 2009 | The Beatles |
| I Don't Like Mondays | The Boomtown Rats |
| People Are Strange | The Doors |
| Riders on the Storm | The Doors |
| The Crystal Ship | The Doors |
| I Hear A Symphony | The Isley Brothers |
| Sunny Afternoon - Remastered | The Kinks |
| Waterloo Sunset | The Kinks |
| The Lonely Shepherd | Gheorghe Zamfir |
| Nights In White Satin | The Moody Blues |
| In the Dutch Mountains | Nits |

Classical

| Elgar: Salut d'amour | Edward Elgar |
| --- | --- |
| Violin Concerto in B Minor, Op. 61: II. Andante | Edward Elgar |
| Nocturne No. 1 in B-Flat Minor, Op. 9 No. 1 - Pt. 1 | Frédéric Chopin |
| Nocturne Op. 9 No. 2 | Frédéric Chopin |
| Nocturne No.20 In C Sharp Minor, Op.posth. | Frédéric Chopin |
| Beethoven's Sonata Pathétique (Piano Sonata No. 8 in C minor, Op. 13) by Sue Sobolewski | Catch up with Saint Saviour |
| Piano Sonata No. 14 in C-Sharp Minor, Op. 27, No. 2 "Moonlight": I. Adagio sostenuto | Ludwig van Beethoven |
| Piano Sonata No. 14 in C-Sharp Minor, Op. 27, No. 2 "Moonlight": II. Allegretto | Ludwig van Beethoven |
| Humoresque, Op. 101, No. 7 | Antonín Dvořák |
| Symphony No. 9 in E Minor, Op. 95, B. 178 "From the New World": II. Largo | Antonín Dvořák |
| Lieder ohne Worte, Op. 19, No. 1 | Felix Mendelssohn |
| Lieder ohne Worte, Op. 19: No. 3 in A (Molto allegro), MWV U 89 - "Hunting Song" | Felix Mendelssohn |
| Lieder ohne Worte, Op. 19: No. 6 in G Minor (Andante sostenuto) "Venetian Gondola Song", MWV U78 | Felix Mendelssohn |
| Lieder ohne Worte, Book 2, Op. 30: VI. Allegretto tranquillo "Venetianisches Gondellied", MWV U110 | Felix Mendelssohn |
| Lieder ohne Worte, Op. 38: No. 6, Andante con moto "Duetto" | Felix Mendelssohn |
| Méditation from Thaïs | Jules Massenet |
| Goldberg-Variationen, BWV 988: I. Aria | Johann Sebastian Bach |
| Goldberg-Variationen, BWV 988: II. Var. I | Johann Sebastian Bach |
| Ave Maria | Johann Sebastian Bach |
| Air on a G String (from Suite No. 3, BWV 1068) | Johann Sebastian Bach |
| Debussy: Arabesque No. 1 | Daniel Pollack |
| Claire de lune | Claude Debussy |
| Preludes, Op. 28: No. 15, Sostenuto in D-Flat Major "Raindrop" | Frédéric Chopin |
| Piano Concerto No. 1 in E Minor, Op. 11: II. Romance. Larghetto | Frédéric Chopin |
| Nocturnes, Op. 27: No. 2, Lento sostenuto in D-Flat Major | Frédéric Chopin |
| Cello Concerto in G Minor, RV 417: II. Andante | Antonio Vivaldi |
| Cello Concerto in A Minor, RV 420: I. Andante | Antonio Vivaldi |
| Piano Sonata No. 11 in A Major, K. 331: I. Theme and Variations | Wolfgang Amadeus Mozart |
| Piano Sonata No. 16 in C Major, K. 545 "Sonata facile": I. Allegro | Wolfgang Amadeus Mozart |
| Piano Sonata No. 16 in C Major, K. 545 "Sonata facile": II. Andante | Wolfgang Amadeus Mozart |
| Piano Concerto No. 21 in C Major, K. 467: II. Andante | Wolfgang Amadeus Mozart |
| 4 Impromptus, Op. 142, D. 935: No. 2 in A-Flat Major: Allegretto | Franz Schubert |
| Impromptu in G-Flat Major, Op. 90 No. 3 (D. 899) | Franz Schubert |
| Carnival of the Animals: The Swan | Camille Saint-Saëns |
| Meditation From Thais | Jules Massenet |
| Schumann - Traumerei | Martin Bloch |
| Schumann Fantasiestücke, Op. 12 I. Des Abends | Classical Piano Academy |
| Arabeske in C, Op. 18 | Robert Schumann |
| Kinderszenen, Op. 15: 1. Von fremden Ländern und Menschen | Robert Schumann |
| 5 Nocturnes | Erik Satie |
| 3 Gymnopédies: No. 1 | Erik Satie |
| Liszt: Consolation No. 3 | Tzimon Barto |
| Canon in D | Johann Pachelbel |
| Cello Suite No. 1 in G Major, BWV 1007: I. Prélude | Johann Sebastian Bach |
| 3 intermezzi, Op. 117: No. 1, Intermezzo in E-Flat Major. Andante moderato | Johannes Brahms |
| 6 Piano Pieces, Op. 118: No. 2, Intermezzo in A Major | Johannes Brahms |
| Walzer Op. 39 Nr. 15, A Major | Johannes Brahms |
| Waltz, Op. 64 No. 2 in C-Sharp Minor - Remastered | Frédéric Chopin |
| Waltz Op. 69 No. 2 | Frédéric Chopin |
| La Moldau | Bedřich Smetana |
| Tchaikovsky Song Without Words, Op.2 No.3: Peter I. Tchaikovsky | Mela Tenenbaum |
| Peer Gynt Suite No. 1, Op. 46: I. Morning Mood | Edvard Grieg |
| Peer Gynt Suite No. 2, Op. 55: IV. Solvejg's Song | Edvard Grieg |
| Lyric Pieces, Op. 12: No. 1, Arietta | Edvard Grieg |
| Preludes, Op. 28: No. 4, Largo in E Minor | Frédéric Chopin |
| Mozart: Piano Trio No. 3 in B-Flat Major, Op. 15 No. 1, K. 502: II. Larghetto | Wolfgang Amadeus Mozart |
| Clarinet Concerto in A Major, K. 622: II. Adagio | Wolfgang Amadeus Mozart |
| Fantasie-impromptu in C-Sharp Minor, Op. 66 | Frédéric Chopin |

Dutch

| Een oude zeeman | Marco de Hollander |
| --- | --- |
| Hoe Je Heette Dat Ben Ik Vergeten | Eddy Christiani |
| Vaarwel Mijn Liefste | Eddy Christiani |
| Zij Gelooft In Mij | Andre Hazes |
| Kleine Jongen | Andre Hazes |
| Ben Ik Te Min | Armand |
| Het Land Van Maas En Waal | Boudewijn de Groot |
| Jimmy | Boudewijn de Groot |
| Prikkebeen | Boudewijn de Groot |
| Testament | Boudewijn de Groot |
| Verdronken Vlinder | Boudewijn de Groot |
| Waterdrager | Boudewijn de Groot |
| Een Roosje, M'n Roosje | Conny Vandenbos |
| Huilen Is Voor Jou Te Laat | Corry Konings |
| Als De Klok Van Arnemuiden | De Havenzangers |
| Greetje Uit De Polder | De Havenzangers |
| Jouw ogen kan ik niet vergeten | De Spelbrekers |
| Katinka | De Spelbrekers |
| Aan De Voet Van Die Oude Wester | De Straatzangers |
| Met Zulke Rozen | Dorus |
| Twee Motten | Dorus |
| Heb Je Even Voor Mij | Frans Bauer |
| Voor Haar | Frans Halsema |
| Kon Ik Maar Even Bij Je Zijn | Gordon |
| Brabant | Guus Meeuwis |
| Het Is Een Nacht... (Levensecht) | Guus Meeuwis |
| Marie, als ik je zie | Heintje Davids |
| Naar De Speeltuin | Heleentje van Capelle |
| Tulpen Uit Amsterdam | Herman Emmink |
| Anne | Herman van Veen |
| Een Vriend Zien Huilen | Herman van Veen |
| Liefde Van Later | Herman van Veen |
| Suzanne | Herman van Veen |
| Heideroosje | Orkest Zonder Naam |
| Als De Morgen Is Gekomen | Jan Smit |
| Dan Volg Je Haar Benen | Jan Smit |
| Ik Zing Dit Lied Voor Jou Alleen | Jan Smit |
| Bij Ons In De Jordaan | Johnny Jordaan |
| Oh, Waterlooplein | Amsterdam Band |
| Ik Zou Wel Eens Willen Weten | Jules de Corte |
| You'll Never Walk Alone | Lee Towers |
| Josje | Ramses Shaffy |
| Laat Me | Ramses Shaffy |
| Sammy | Ramses Shaffy |
| Wij Zullen Doorgaan - 1975 Single Version | Ramses Shaffy |
| Zing-Vecht-Huil-Bid-Lach-Werk En Bewonder | Ramses Shaffy |
| Als de Dag van Toen | Reinhard Mey |
| Gute Nacht, Freunde | Reinhard Mey |
| Banger Hart | Rob De Nijs |
| Malle Babbe | Rob De Nijs |
| De Zuiderzee Ballade | Sylvain Poons |
| Diep In Mijn Hart | Tante Leen |
| Oh Johnny | Tante Leen |
| N Beetje | Teddy Scholten |
| Vierentwintig Rozen | Toon Hermans |
| Bedankt Lieve Ouders | Vader Abraham |
| 't Kleine Café Aan De Haven | Vader Abraham |
| Daar Bij De Waterkant | Black & White |
| Ome Jan | Willeke Alberti |
| Droomland | Willy Alberti |
| Aan De Amsterdamse Grachten | Wim Sonneveld |
| Annemarie (Ma Petite Chanson) - Live | Wim Sonneveld |
| Een Zwoele Nacht In Krimpen Aan De IJssel | Wim Sonneveld |
| Het Dorp | Wim Sonneveld |
| Huwelijksreis (Le Voyage De Noces) - Live | Wim Sonneveld |
| Ik Heb Zo Vaak Aan Amsterdam Gedacht | Wim Sonneveld |
| Margootje | Wim Sonneveld |
| Verliefd Op Juffrouw Van Dam | Wim Sonneveld |
| Marjolijne | Wim Sonneveld |
| Ach Vaderlief | Zangeres Zonder Naam |
| Oude man met je akkordeon | Zwarte Riek |

Jazz & Blues

| Route 66 | Nat King Cole |
| --- | --- |
| All Your Love - Stereo | Sam Maghett |
| At Last | Etta James |
| Autumn Leaves | Bill Evans |
| Baby Get Lost | Dinah Washington |
| Bird Of Paradise | Charlie Parker |
| Black And Blue | Louis Armstrong |
| Blue in Green (feat. John Coltrane & Bill Evans) | Miles Davis |
| C & A Blues | Big Bill Broonzy |
| Chain Gang Blues | Champion Jack Dupree |
| Come Fly With Me - 2008 Remastered | Frank Sinatra |
| Crawlin' King Snake | John Lee Hooker |
| Don't Let Me Be Misunderstood | Nina Simone |
| Satin Doll | Duke Ellington |
| Every Day I Have the Blues - Remastered | B.B. King |
| Go Down Sunshine | Cuby & The Blizzards |
| God Bless The Child | Billie Holiday |
| Guess Who | B.B. King |
| Gypsy Woman | Muddy Waters |
| Heavy Heart Blues | Champion Jack Dupree |
| I Can't Get Started - 2001 Remastered Version | Dizzy Gillespie |
| I Fall In Love Too Easily | Chet Baker |
| I Feel Like Going Home | Muddy Waters |
| I Hear A Symphony | The Isley Brothers |
| I Heard It Through The Grapevine | Marvin Gaye |
| I Loves You Porgy - 2013 Remastered Version | Nina Simone |
| I Only Have Eyes For You | Coleman Hawkins |
| I'm Drinking My Whisky | Cuby & The Blizzards |
| I'm In The Mood | John Lee Hooker |
| I've Got You Under My Skin | Frank Sinatra |
| Just the Two of Us (feat. Bill Withers) - Edit | Grover Washington, Jr. |
| Kind Hearted Woman | Muddy Waters |
| Lovely Day | Bill Withers |
| Lover Man | Sarah Vaughan |
| Lullaby of Birdland | Dexter Gordon |
| Me and My Gin | Bessie Smith |
| Mood Indigo | Duke Ellington |
| Moonlight In Vermont | Gerry Mulligan Quartet |
| My Baby Just Cares for Me | Nina Simone |
| My Funny Valentine | Chet Baker |
| My Girl | The Temptations |
| Nature Boy | Stan Getz |
| Nobody Knows You When You're Down and Out | Bessie Smith |
| Green Dolphin Street | Teddy Wilson |
| Out Of Nowhere | Ella Fitzgerald |
| Poor Man's Blues | Bessie Smith |
| Sing Me A Swing Song (And Let Me Dance) | Ella Fitzgerald |
| (Sittin' On) the Dock of the Bay | Otis Redding |
| So What | Miles Davis |
| Sophisticated Lady | Al Jarreau |
| St. Louis Blues | Bessie Smith |
| Strangers In The Night | Frank Sinatra |
| Summertime | Miles Davis |
| Summertime | Charlie Parker |
| Sunny | Bobby Hebb |
| Take Five | The Dave Brubeck Quartet |
| The Girl Next Door | Wes Montgomery |
| The Thrill Is Gone | B.B. King |
| They Can't Take That Away From Me | Carmen Cavallaro |
| Time After Time | Chet Baker |
| Unchain My Heart | Ray Charles |
| Unchained Melody | The Righteous Brothers |
| What A Wonderful World | Louis Armstrong |
| What's Going On | Marvin Gaye |

**Supplementary table S3. Logistic Regression Model**

| **Variables** | **B** | **SE** | **Wald** | **P-Value** | **OR** | **95% Confidence interval** | |
| --- | --- | --- | --- | --- | --- | --- | --- |
|  |  |  |  |  |  | Lower | Upper |
| **Music group** | -0,838 | 0,419 | 4,003 | 0,045 | 0,433 | 0,190 | 0,983 |
| **Age** | 0,050 | 0,028 | 3,198 | 0,074 | 1,051 | 0,995 | 1,110 |
| **Delirium prior to admission** | 0,821 | 0,503 | 2,666 | 0,103 | 2,272 | 0,848 | 6,085 |
| **Cognitive impairment** | 1,541 | 0,430 | 12,871 | <0,001 | 4,671 | 2,012 | 10,842 |
| **ASA ≥ 3** | 1,080 | 0,474 | 5,199 | 0,023 | 2,944 | 1,164 | 7,450 |
| **Constant** | -7,622 | 2,309 | 10,901 | <0,001 | 0,000 |  |  |

B: regression coefficient; SE: standard error; Wald: Wald test statistic; *p*: significance level; OR: odds ratio (Exp(B)). The constant (intercept) represents the log odds of postoperative delirium when all predictor variables are set to zero. Statistical significance was defined as *p* <0.05.

**Supplementary table S4. First day of postoperative delirium**

|  | **Intention-to-treat population (ITT)*** | | **Modified Intention-to-treat population (mITT)*** | |
| --- | --- | --- | --- | --- |
| **Initial postoperative day of delirium:** | Control (n=223) | Music (n=226) | Control (n=215) | Music (n=202) |
| 1 | 20 (9) | 11 (4.9) | 20 (9.3) | 8 (4.0) |
| 2 | 7 (3.1) | 3 (1.3) | 7 (3.3) | 2 (1.0) |
| 3 | 1 (0.4) | 3 (1.3) | 1 (0.5) | 2 (1.0) |
| 4 | 0 (0) | 2 (0.9) | 0 (0) | 1 (0.5) |
| 5 | 0 (0) | 2 (0.9) | 0 (0) | 1 (0.5) |
| 6 | 0 (0) | 0 (0) | 0 (0) | 0 (0) |
| 7 | 1 (0.4) | 0 (0) | 1 (0.5) | 0 (0) |
| **No delirium** | 194 (87) | 205 (90.7) | 186 (86.5) | 188 (93.1) |

**Supplementary figure S5. Fidelity of music intervention**

|  | **Pre-operative** | | **Intra-operative** | | **POD-1 Morning (1M)** | | **POD-1 Evening (1E)** | | **POD-2 Morning (2M)** | | **POD-2 Evening (2E)** | | **POD-3 Morning (3M)** | | **POD-3 Evening (3E)** | | **POD-4 Morning (4M)** | | **POD-4 Evening (4E)** | | **POD-5 Morning (5M)** | | **POD-5 Evening (5E)** | |
| --- | --- | --- | --- | --- | --- | --- | --- | --- | --- | --- | --- | --- | --- | --- | --- | --- | --- | --- | --- | --- | --- | --- | --- | --- |
|  | *N* | %* | *N* | %* | *N* | %* | *N* | %* | *N* | %* | *N* | %* | *N* | %* | *N* | %* | *N* | %* | *N* | %* | *N* | %* | *N* | %* |
| **Yes** | 142 | 70 | 126 | 62 | 110 | 55 | 99 | 51 | 91 | 48 | 87 | 47 | 74 | 43 | 64 | 39 | 64 | 42 | 53 | 37 | 46 | 35 | 45 | 35 |
| **No** | 26 | 13 | 38 | 19 | 41 | 21 | 44 | 22 | 37 | 19 | 42 | 22 | 44 | 25 | 45 | 27 | 31 | 21 | 36 | 25 | 31 | 24 | 28 | 22 |
| **Unknown** | 34 | 17 | 38 | 19 | 45 | 23 | 46 | 23 | 51 | 27 | 46 | 25 | 42 | 24 | 41 | 25 | 41 | 27 | 41 | 28 | 39 | 30 | 41 | 32 |
| **Early withdrawal** | 0 | 0 | 0 | 0 | 4 | 2 | 7 | 4 | 11 | 6 | 12 | 6 | 13 | 8 | 14 | 9 | 15 | 10 | 15 | 10 | 15 | 11 | 15 | 12 |
| **Discharged** | 0 |  | 0 |  | 2 |  | 6 |  | 12 |  | 15 |  | 28 |  | 38 |  | 51 |  | 57 |  | 71 |  | 73 |  |
| **Total** | 202 |  | 202 |  | 202 |  | 202 |  | 202 |  | 202 |  | 202 |  | 202 |  | 202 |  | 202 |  | 202 |  | 202 |  |

Patients of music group in the mITT population. *Percentage of participating patients. 1 M: postoperative day 1 in the morning, 1 E: postoperative day 1 in the evening, 2 M: postoperative day 1 in the morning and so on.

**Supplementary table S6. Identification of delirium**

| **Postoperative day** | **N total participants** | **DOS assessment** | **Elevated DOS** | **Assessment of geriatician after elevated DOS** |
| --- | --- | --- | --- | --- |
|  |  | N (%) | N | N (%) |
| 1 | 449 | 375 (83.5) | 50 | 38 (76) |
| 2 | 439 | 334 (76.1) | 38 | 27 (71.1) |
| 3 | 423 | 276 (65.2) | 33 | 25 (75.8) |
| 4 | 404 | 210 (51.9) | 14 | 11 (78.6) |
| 5 | 343 | 183 (53.4) | 9 | 4 (44.4) |
| 6 | 288 | 107 (37.2) | 11 | 8 (72.7) |
| 7 | 238 | 66 (27.7) | 7 | 4 (57.1) |
