# Supplementary material for: Effect of perioperative music on delirium after hip fracture operations (MCHOPIN): a multicentre randomised clinical trial in Dutch hospitals
Source: BMJ Open. 2025 Aug 28;15(8):e095819. doi: 10.1136/bmjopen-2024-095819 (PMC12406818; doi:10.1136/bmjopen-2024-095819)
Supplement: Supplementary file 2 [file bmjopen-2024-095819.reviewer_comments.pdf]

PEER REVIEW HISTORY

BMJ Open publishes all reviews undertaken for accepted manuscripts. Reviewers are asked to complete a checklist review form and are provided with free text boxes to elaborate on their assessment. These free text comments are reproduced below.

ARTICLE DETAILS

Title (Provisional)

Effect of perioperative MusiC on delirium after Hip fracture OPeratIoNs (MCHOPIN): a multicentre randomised clinical trial in Dutch hospitals

Authors

Dirven, Thomas Lodewijk Adriaan; Fu, Victor X.; Becker, Antonia S.; Andrinopoulou, Eleni-Rosalina; Kalisvaart, Kees; Jeekel, Johannes; Klimek, M; Verhofstad, Michael H.J.; Van der Velde, Detlef; Slegers, Leonie; Haverlag, Robert; Haumann, Johan; Heemskerk, Johan; van der Elst, Maarten; Hartholt, Klaas; van Cosburgh, Jurjan; Wörner, Lisa; Aukema, Tjeerd S.; Willems, Siem; Greeven, Alexander P. A.; Romsom, Jesse; Leijnen, Michiel; van Schie, Peter; Spierenburg, Geert; van der Bij, Gerben; Boom, Annemieke; ter Riet, Stefan; van Duijvenvoorde, Erik; Rad, Mandana; Hoencamp, Rigo; van Noort, A; van Boekel, Anniek; Berger, Isabel

---

VERSION 1 - REVIEW

Reviewer 1

Name Huang, Lining

Affiliation The Second Hospital of Hebei Medical University, Department of Anesthesiology

Date 17-Mar-2025

COI None

Dear Authors,

Thank you for choosing BMJ Open to publish your manuscript. Here below, I have provided some suggestions.

Introduction:

I noticed that you attempted to illustrate the innovation and necessity of this study through a meta-analysis. However, you did not specifically delineate the gaps compared to other literature. Please emphasize in the introduction what innovative aspects this study offers in comparison to other relevant research.

Method:

1.The inclusion criteria for this study were surgical patients with proximal femoral fractures aged ≥65 years. The inclusion criteria appear to be simplistic and broad. For instance, is the patient's ASA classification one of the inclusion criteria? Hip fracture patients are predominantly elderly individuals who may have pre-existing cognitive impairments or psychiatric disorders. These factors can significantly impact the incidence of postoperative delirium. Please specify whether such patients were excluded from the study.

2.Please elucidate the genres of the music, such as pop, classical, or other categories, as the characteristics of the tonal properties may exert a certain influence on the affective state of the patients.

3.Please provide additional details regarding the anesthesia and intraoperative conditions, such as the method of anesthesia, the anesthetic agents administered, and the management of hemodynamic instability during the procedure.

4.The heterogeneity of postoperative ward environments may exert a significant confounding effect on the efficacy of music interventions. Specifically, variables such as ambient noise levels, ward type (single room vs. multi-bed room), and visitor flow may act as moderating variables that influence the effectiveness of music interventions. The variability in these environmental factors could lead to heterogeneity of treatment effect, thereby compromising the internal validity of the study findings. To ensure the consistency of music intervention outcomes, we recommend providing a detailed description of environmental control measures.

5.The evaluation of DSM-V must be conducted by a psychiatrist or a trained clinician, which may limit its applicability in clinical settings. Diagnosing patients with mild or atypical symptoms can be particularly challenging. The assessment under DSM-V typically requires detailed history taking, physical examination, and cognitive evaluation, which can be time-consuming, especially in busy clinical environments. In clinical settings where rapid identification of delirium is necessary, such as in emergency departments or postoperative wards, DSM-V may not be sufficiently practical. Please specifically describe the two methods mentioned for diagnosing delirium, the DOS-score and DSM-V. Additionally, as the Confusion Assessment Method (CAM) is a commonly used clinical tool for diagnosing delirium, why was CAM not chosen as the assessment method in this study?

6.The results included an assessment of anxiety; however, the methods section did not mention the specific approach used to evaluate anxiety. Please provide details on how anxiety was assessed using the State-Trait Anxiety Inventory.

Results:

1.Please revise the tables in the manuscript to conform to the standard three-line table format.

2.Please specify which variables were adjusted for in the logistic regression results.

3.The variables included in the logistic regression model are as follows: age, ASA ≥ 3, pre-admission delirium, and cognitive decline indicated by the Mini-Cog. Was intraoperative hypotension accounted for in the analysis? Both the occurrence and duration of intraoperative hypotension could potentially be influencing factors for postoperative delirium. Should these be incorporated into the model for analysis? Additionally, preoperative anxiety is also considered a risk factor for postoperative delirium. Was the preoperative anxiety status analyzed?

4.Please provide accurate descriptions of the titles for Table 2 and Table 3 in accordance with the statistical methods and results.

Discussion and Conclusion:

1.The discussion section contains an excessive focus on patient compliance issues. Since the primary outcome of this study is the effect of perioperative music listening on postoperative delirium, it is recommended to explore the potential mechanisms by which music may influence postoperative delirium and to compare these findings with those of other studies.

**Reviewer**	**2**
**Name**	**Keage, Hannah**
**Affiliation**	**University of South Australia Division of Health Sciences**
**Date**	**14-May-2025**
**COI**	**None**

Thank you for asking me to review this manuscript. It was well written and formatted, however I do have some comments to be addressed before it’s published.

Major

- How did the presence of delirium affect the implantation of the music intervention post operatively? It’s stated that post operatively, the intervention was provided daily two times for 30 minutes. Was this more problematic to implement in those with delirium, especially those with hyperactive symptoms? Sorry if I missed it, but I couldn’t see reports of the % of music sessions implemented/day/group.

- Was the delirium assessment undertaken everyday, or just on weekdays, and was it (roughly) at the same time of day? The DOS was no performed in 17-35% of cases within the first three days; that is a lot of missing data. The delirium incidence rate was low, and I think these methodological issues contributed, and should be noted more so in the limitations. Could sensitivity analysis be run to assess effects in those systematically and comprehensively assessed for the presence/absence of delirium (in first three days, for example)?

- The protocol was unreferenced ("In the previous published protocol….", page 10).

- How was previous delirium (prior to admission) measured? Clinical or hospital notes? How many had this recorded?

- Please report the full (primary outcome) logistic regression models with covariates; currently, it's impossible to know how the covariates contributed to the outcome (delirium), as only the effect of the intervention is reported.

- Please integrate effect sizes into the Discussion - e.g. "a positive effect", "a significant decrease" - should be accompanied by the effect size.

Minor

- The term older is typically preferred to elderly.

- Cognitive decline is listed as a covariate, is this meant to be cognitive impairment (as per Table 1)? Decline would need >1 measurement over time.

VERSION 1 - AUTHOR RESPONSE

Reviewer 1: Comments to author
Dr. Lining Huang, The Second Hospital of Hebei Medical University
Introduction:

1. I noticed that you attempted to illustrate the innovation and necessity of this study through a meta-analysis. However, you did not specifically delineate the gaps compared to other literature. Please emphasize in the introduction what innovative aspects this study offers in comparison to other relevant research.
   Our response: Thank you for mentioning the need of additional information on this topic in the introduction. We have added additional information in the introduction to emphasize the innovative aspects of this study.
   See introduction: 'A recent meta-analysis (1) found that music interventions may be effective in reducing delirium. However, the included studies showed substantial heterogeneity and the sample sizes were relatively small (n=22-147), particularly in studies focusing on patients undergoing hip surgery. Also, none of the studies used a delirium diagnosis based on DSM-V criteria. This study addresses these gaps by conducting a multicentre randomised clinical trial in a substantial population of elderly hip fracture patients, using DSM-V criteria.

   The objective of this trial was to investigate the effect of perioperative music, i.e. music before, during and after surgery, on incidence of postoperative delirium in elderly hip fracture patients.'

Method:

2. The inclusion criteria for this study were surgical patients with proximal femoral fractures aged ≥ 65 years. The inclusion criteria appear to be simplistic and broad.

For instance, is the patient's ASA classification one of the inclusion criteria? Hip fracture patients are predominantly elderly individuals who may have pre-existing cognitive impairments or psychiatric disorders. These factors can significantly impact the incidence of postoperative delirium. Please specify whether such patients were excluded from the study.

Our response: We did not apply inclusion or exclusion criteria based on ASA classification, as our aim was to include a representative sample of the real-world population admitted for hip fracture surgery. A large multicentre study by Johansen et al. (2), which analysed 59,369 hip fracture patients in the UK, showed that only 28.6% of patients fell into ASA class I or II, while the majority were classified as ASA III (56.8%) or ASA IV (14.1%). For instance, if we would restrict our inclusion to <ASA 3, this would exclude around 70% of the patient population, limiting the generalisability of our findings. Additionally, due to the large sample size and standardized randomisation procedure, we expect that pre-existing cognitive impairments and psychiatric disorders were evenly distributed across both randomisation groups.

We acknowledge that ASA classification, as well as pre-existing cognitive impairment and psychiatric disorders, are important risk factors for postoperative delirium. Therefore, we adjusted for ASA classification, age, pre-existing cognitive decline, and delirium prior to admission in our multivariable logistic regression model to account for these potential confounders.

We have added a part in our discussion to describe this in our manuscript: 'We believe we included a representative sample of hip fracture patients. To enhance generalisability, we did not apply ASA-based exclusion criteria. Potential confounders such as ASA classification and prior cognitive impairment were adjusted for in our regression model, and we expect an even distribution of cognitive and psychiatric conditions due to randomisation and large sample size.'

3. Please elucidate the genres of the music, such as pop, classical, or other categories, as the characteristics of the tonal properties may exert a certain influence on the affective state of the patients.

   Our response: We thank the reviewer for this thoughtful and relevant comment. The music intervention consisted of four preselected playlists, categorized by genre: classical, jazz and blues, pop, and Dutch music. The selection was curated by a panel of five research physicians with substantial expertise in perioperative music, based on literature recommendations and music used in previous studies. The repertoire primarily included popular music from the 1950s to 1980s—eras corresponding to the patients' youth and early adulthood—to enhance familiarity, as familiar auditory environments have been associated with a reduced risk of delirium (3).

   Patients were encouraged to choose music from these playlists or select music via Spotify, enabling patients to tailor the music to their preferences. While the exact mechanism by which music may prevent delirium remains unclear (see also our

response to point 16), it is not yet established whether specific tonal properties or genres have a better effect on preventing delirium.
A recent large randomized experimental study (4) investigated the effect of music: listening to a specific genre (urban, electronic, classical, rock, or pop) vs. listening to a preferred genre. The authors found that listening to a specific genre was not associated with increased pain tolerance, while listening to a personally preferred genre did lead to increased pain tolerance. These findings highlight the importance of individualized music preferences over the influence of universal tonal properties, supporting our approach to offer personalized music options.
We have described this in our manuscript in the method section: 'It remains unclear whether specific genres are more effective in preventing delirium. However, prior research suggests that personalized music is more effective than fixed genres (31), which supports our use of patient-preferred music.'

4. Please provide additional details regarding the anaesthesia and intraoperative conditions, such as the method of anaesthesia, the anaesthetic agents administered, and the management of hemodynamic instability during the procedure.
   Our response: Thank you for your valuable comment regarding the anaesthesia and intraoperative conditions.
   In line with routine clinical practice, the choice of anaesthesia—general or locoregional—was left to the discretion of the attending anaesthesiologist and surgical team. As shown in Table 1 (Baseline Characteristics), the types of anaesthesia administered included spinal anaesthesia (n=228, 49%), intravenous anaesthesia (n=186, 40%), and inhalational anaesthesia (n=51, 11%). Additionally, 218 patients (48.6%) received a peripheral nerve block, with comparable distribution between the music and control groups (110 vs. 108).
   Further details on the anaesthesia protocol and intraoperative management are described in our previously published study protocol (5). But we have also added this text in the manuscript: 'Anaesthesia depth was monitored using BIS or equivalent devices where available. Despite evidence suggesting music may reduce propofol requirements (22), during hip fracture surgery most centres used volatile agents, and sedative dosages were not systematically recorded.'
   We have also added this part in our discussion, see: 'Given the complex aetiology and the multitude of factors that may contribute to postoperative delirium (6), we did not adjust for all potential confounding variables, such as intraoperative hypotension (7), which may have influenced our findings. However, we believe that, given our large sample size and standardized randomisation procedure, confounding factors are evenly distributed across both groups.'

5. The heterogeneity of postoperative ward environments may exert a significant confounding effect on the efficacy of music interventions. Specifically, variables such as ambient noise levels, ward type (single room vs. multi-bed room), and visitor flow may act as moderating variables that influence the effectiveness of music interventions. The variability in these environmental factors could lead to heterogeneity of treatment effect, thereby compromising the internal validity of the

study findings. To ensure the consistency of music intervention outcomes, we recommend providing a detailed description of environmental control measures.

Our response: Thank you for this important observation regarding the potential confounding influence of postoperative ward environments on the effectiveness of music interventions.

While efforts were made to provide patients with as much privacy as possible, including placement in single rooms when available, logistical constraints made it unfeasible to guarantee single-room accommodation for all participants. As a result, some patients were accommodated in multi-bed rooms. To minimize environmental variability during the intervention, music sessions were scheduled during quieter periods of the day—typically after medical rounds and in the evening after visiting hours—when ambient noise and visitor presence were expected to be lower. Unfortunately, due to the complex logistics of this multicentre study, we were unable to systematically record the ambient noise levels or specific ward locations during the intervention periods. We agree that environmental factors may have introduced heterogeneity in treatment effect. Nevertheless, the pragmatic design of our study reflects real-world clinical conditions, which enhances the external validity of our findings. We have added a description about this subject in our discussion, see: 'Due to hospital logistics, patients could not consistently be accommodated in single rooms, potentially introducing variability in environmental factors such as ambient noise. Given the complex aetiology and the multitude of factors that may contribute to postoperative delirium (44), we did not adjust for all potential confounding variables, such as intraoperative hypotension (62) and environmental factors. However, we believe that, given our large sample size and standardized randomisation procedure, confounding factors are evenly distributed across both groups.'

6. The evaluation of DSM-V must be conducted by a psychiatrist or a trained clinician, which may limit its applicability in clinical settings. Diagnosing patients with mild or atypical symptoms can be particularly challenging. The assessment under DSM-V typically requires detailed history taking, physical examination, and cognitive evaluation, which can be time-consuming, especially in busy clinical environments. In clinical settings where rapid identification of delirium is necessary, such as in emergency departments or postoperative wards, DSM-V may not be sufficiently practical. Please specifically describe the two methods mentioned for diagnosing delirium, the DOS-score and DSM-V. Additionally, as the Confusion Assessment Method (CAM) is a commonly used clinical tool for diagnosing delirium, why was CAM not chosen as the assessment method in this study?

   Our response: In our study, delirium screening was performed using the Delirium Observation Screening Scale (DOSS), a 13-item nurse-administered tool validated in elderly and hip fracture patients. Our study procedures regarding the DOSS are described in the cited protocol (5), which is referenced in our manuscript.

   The DOSS end score, calculated as the average of three shift-based assessments per day, has shown high sensitivity (94.4%) and acceptable specificity (76.6%) for

detecting delirium (8). In line with the Dutch national guidelines, a DOSS score of ≥3 prompted a clinical evaluation by a geriatrician, who confirmed the diagnosis using DSM-5 criteria. The DOSS is widely implemented in Dutch hospitals and familiar to nursing staff, which facilitates consistent daily screening. Although newer tools such as the 4AT may be less time-consuming, the DOSS remains the preferred instrument in the Netherlands due to its integration in routine care (9). We added this to our method section in the manuscript: 'The DOSS remained the preferred instrument in the Netherlands due to its integration in routine care(9)'
We have also added the following text to our discussion: "In accordance with Dutch guidelines, we used DSM-5 for diagnosing. However, a recent systematic review showed that 3D-CAM offers strong diagnostic accuracy and is less time-consuming. Therefore, we recommend its use in future research to streamline delirium assessment (54)."

7. The results included an assessment of anxiety; however, the methods section did not mention the specific approach used to evaluate anxiety. Please provide details on how anxiety was assessed using the State-Trait Anxiety Inventory.
Our response: Thank you for your comment. Further details on the assessment of anxiety are described in our previously published study protocol (5). Anxiety was measured using the Six-Item State-Trait Anxiety Inventory (STAI-6), a validated short form of the original 20-item STAI-State subscale. The STAI-6 consists of six items rated on a 4-point Likert scale. Scoring is performed by reverse scoring the three positively worded items, summing all six responses, and multiplying the total by 20/6 to yield a final score ranging from 20 to 80, with higher scores indicating greater anxiety. The STAI-6 was administered preoperatively in the emergency department or surgical ward, and postoperatively on days 1 and 2. See the following adjusted text in the manuscript: 'To further explore the impact of music, secondary outcomes were analysed, as described in the previously published protocol (5). Anxiety was measured using the Six-Item State-Trait Anxiety Inventory (STAI-6), a validated short form of the original 20-item STAI-State subscale. The STAI-6 was administered preoperatively in the emergency department or surgical ward, and on postoperative day 1 and 2.'

Results:

8. Please revise the tables in the manuscript to conform to the standard three-line table format.
Our response: Thank you for your comment. The layout of all tables in the manuscript has been revised to conform to the standard three-line table format, in accordance with journal guidelines. We appreciate your attention to detail.
9. Please specify which variables were adjusted for in the logistic regression results.
Our response: The following text from the manuscript elaborates on the variables from the logistic regression model: 'A logistic regression model was developed to correct for possible confounding variables, which were added based on previous

literature (6). The following variables were entered into the model: age, ASA>=3, delirium prior to admission, cognitive impairment according to Mini-Cog.'

10. The variables included in the logistic regression model are as follows: age, ASA ≥ 3, pre-admission delirium, and cognitive decline indicated by the Mini-Cog. Was intraoperative hypotension accounted for in the analysis? Both the occurrence and duration of intraoperative hypotension could potentially be influencing factors for postoperative delirium. Should these be incorporated into the model for analysis? Additionally, preoperative anxiety is also considered a risk factor for postoperative delirium. Was the preoperative anxiety status analyzed?
    Our response: Thank you for your valuable comment, regarding the possible influence of intraoperative hypotension on the postoperative delirium in our studied population. Unfortunately, intraoperative hypotension was not recorded in our dataset, and therefore we were unable to adjust for its occurrence or duration in our analysis. We have added the following sentence to the discussion section to elaborate on intraoperative hypotension as a possible confounding factor: 'Given the complex aetiology and the multitude of factors that may contribute to postoperative delirium (44), we did not adjust for all potential confounding variables, such as intraoperative hypotension (62) and environmental factors. However, we believe that, given our large sample size and standardized randomisation procedure, confounding factors are evenly distributed across both groups.'
    Regarding preoperative anxiety, although this variable was measured using a questionnaire, the number of completed assessments was insufficient to include it reliably in the regression model, as we have highlighted in our discussion: 'Additionally, we believe that limited time among nursing staff contributed to missing data, such as the STAI-6, from the questionnaires (10). Conducting research in frail patients in acute care is challenging, but essential to ensure that study findings are applicable to the target clinical population (11).'
11. Please provide accurate descriptions of the titles for Table 2 and Table 3 in accordance with the statistical methods and results.
    Our response: Thank you for your valuable comment, we have adjusted the titles from both Tables.

Discussion and Conclusion:

12. The discussion section contains an excessive focus on patient compliance issues. Since the primary outcome of this study is the effect of perioperative music listening on postoperative delirium, it is recommended to explore the potential mechanisms by which music may influence postoperative delirium and to compare these findings with those of other studies.
    Our response: Thank you for your valuable feedback. We have revised the discussion section to reduce the emphasis on patient compliance and have instead expanded on the potential mechanisms through which perioperative music listening may reduce the incidence of postoperative delirium.
    See the following part in our discussion: ´Despite our hypothesis and contrary to previous studies (19), no reduction in pain or anxiety was observed. Music might not mitigate delirium by modulating its known risk factors. Although the pathophysiology

of (postoperative) delirium remains incompletely understood, prior research implicates mechanisms such as neuroinflammation and neurotransmitter imbalance (18). Particularly in patients with a hip fracture, pain and surgical stress may induce a systemic release of pro-inflammatory cytokines (18), which can influence the hippocampus and amygdala and activate the sympathetic nervous system (65). This, in turn, activates the hypothalamic–pituitary–adrenal (HPA) axis, resulting in elevated cortisol levels, which have been linked to the development of delirium (66). Music has been hypothesized to induce anti-inflammatory effects by stimulating the parasympathetic nervous system via the vagal nerve, potentially modulating relevant physiological pathways (50). Moreover, music has been associated with reduced serum cortisol levels, which may represent an additional mechanism through which it helps prevent delirium (21). Therefore, we encourage future studies to focus on the mechanism of action of music intervention on delirium to elucidate the preventive effect.'

Reviewer 2: Comments to author

Dr. Hannah Keage, University of South Australia Division of Health Sciences

13. How did the presence of delirium affect the implantation of the music intervention post operatively? It's stated that post operatively, the intervention was provided daily two times for 30 minutes. Was this more problematic to implement in those with delirium, especially those with hyperactive symptoms? Sorry if I missed it, but I couldn't see reports of the % of music sessions implemented/day/group.
    Our response: Thank you for your insightful comment. We have analysed the adherence to the music intervention in patients who developed postoperative delirium compared to those who did not. Interestingly, adherence was 8% higher in the delirium group than in the non-delirium group. Furthermore, 12 out of the 14 patients who developed delirium were diagnosed between postoperative day 1 and 3, with 8 of them diagnosed on the first postoperative day. This suggests that the presence of delirium did not hinder the implementation of the intervention.
    A previous study shows that the hypoactive subtype is more common than the hyperactive or mixed subtype (65.2% vs 7.2% vs 27.6% (12)). This might be an explanation for the easier adherence of the intervention in patients with delirium. However, we would like to emphasize that the primary aim of our study was to evaluate whether perioperative music could reduce the incidence of postoperative delirium. The implementation of the intervention in patients already diagnosed with delirium was not a primary focus of our analysis.
    Details on the fidelity of the music intervention, including adherence rates per day, are presented in 'Supplementary Figure S4. Fidelity of Music Intervention'.
14. Was the delirium assessment undertaken everyday, or just on weekdays, and was it (roughly) at the same time of day? The DOS was no performed in 17-35% of cases within the first three days; that is a lot of missing data. The delirium incidence rate was low, and I think these methodological issues contributed, and should be noted more so in the limitations. Could sensitivity analysis be run to assess effects in those

systematically and comprehensively assessed for the presence/absence of delirium (in first three days, for example)?

Our response: Thank you for your thoughtful comment. The delirium assessments were intended to be conducted daily, approximately at the same time of day, during one of the three nursing shifts (morning, evening, or night). However, as noted, there was a substantial proportion of missing DOS assessments (17–35%) within the first three postoperative days.

To address this concern, we performed a sensitivity analysis within the modified intention-to-treat (mITT) population, including only patients for whom more than 60% of the DOS assessments were completed during the first three postoperative days. In this subgroup, the incidence of postoperative delirium was 14.9% in the control group and 9.6% in the music intervention group.

We agree that the missing data may have influenced the observed incidence of delirium. We have now emphasized this limitation more clearly in the revised discussion section.

15. The protocol was unreferenced ("In the previous published protocol….", page 10).

    Our response: Thank you for this important observation. We have added the reference of our protocol on page 10.

16. How was previous delirium (prior to admission) measured? Clinical or hospital notes? How many had this recorded?

    Our response: Thank you for your question about the incidence of delirium prior to admission. The delirium incidence was measured using clinical notes. Fourty-two participants (9.4%) had a delirium prior to admission. Delirium is also often under-recognized, specifically in outpatients, as the symptoms can overlap with symptoms of other diseases, such as dementia. Addessi et al. (2018) found that 13% had delirium of elderly outpatients with dementia (13). Patients with dementia have an increased risk on developing delirium. Therefore, we believe that our measured delirium prior to admission is comparable to other studies.

17. Please report the full (primary outcome) logistic regression models with covariates; currently, it's impossible to know how the covariates contributed to the outcome (delirium), as only the effect of the intervention is reported.

    Our response: Thank you for suggesting improvements for the presentation of the logistic regression model. We have added the logistic regression model in supplementary table S3.

18. Please integrate effect sizes into the Discussion - e.g. "a positive effect", "a significant decrease" - should be accompanied by the effect size.

    Our response: Thank you for the suggestion. We have added the effect sizes in several parts in de discussion.

Minor

19. The term older is typically preferred to elderly.

    Our response: Thank you for the suggestion. We have replaced the term "elderly" throughout the manuscript to align with current preferred terminology.

20. Cognitive decline is listed as a covariate, is this meant to be cognitive impairment (as per Table 1)? Decline would need >1 measurement over time.

Our response: Thank you for the good suggestion to improve our manuscript. We have adjusted the manuscript.

References:

1. Golubovic J, Neerland BE, Aune D, Baker FA. Music Interventions and Delirium in Adults: A Systematic Literature Review and Meta-Analysis. Brain Sci. 2022;12(5).
2. Johansen A, Tsang C, Boulton C, Wakeman R, Moppett I. Understanding mortality rates after hip fracture repair using ASA physical status in the National Hip Fracture Database. Anaesthesia. 2017;72(8):961-6.
3. Meagher DJ. Delirium: optimising management. Bmj. 2001;322(7279):144-9.
4. Van der Valk Bouman ES, Becker AS, Schaap J, Berghman M, Oude Groeniger J, Van Groeningen M, et al. The impact of different music genres on pain tolerance: emphasizing the significance of individual music genre preferences. Sci Rep. 2024;14(1):21798.
5. Fu VX, Jeekel J, Van Lieshout EMM, Van der Velde D, Slegers LJP, Haverlag R, et al. Effect of music on clinical outcome after hip fracture operations (MCHOPIN): study protocol of a multicentre randomised controlled trial. BMJ Open. 2021;11(12):e049706.
6. Qi Y-m, Li Y-j, Zou J-h, Qiu X-d, Sun J, Rui Y-f. Risk factors for postoperative delirium in geriatric patients with hip fracture: A systematic review and meta-analysis. Frontiers in Aging Neuroscience. 2022;14.
7. Radinovic K, Markovic Denic L, Milan Z, Cirkovic A, Baralic M, Bumbasirevic V. Impact of intraoperative blood pressure, blood pressure fluctuation, and pulse pressure on postoperative delirium in elderly patients with hip fracture: A prospective cohort study. Injury. 2019;50(9):1558-64.
8. Schuurmans MJ, Shortridge-Baggett LM, Duursma SA. The Delirium Observation Screening Scale: a screening instrument for delirium. Res Theory Nurs Pract. 2003;17(1):31-50.
9. Richtlijnendatabase. Instruments for screening and diagnosis of delirium Utrecht: Federatie Medisch Specialisten; 2024 [Available from: https://richtlijnendatabase.nl/richtlijn/delier_bij_volwassenen_en_ouderen/meetinstrumenten_voor_screening_en_diagnostiek_van_delier.html.
10. Sparano F, Aaronson NK, Sprangers MAG, Fayers P, Pusic A, Kieffer JM, et al. Inclusion of older patients with cancer in randomised controlled trials with patient-reported outcomes: a systematic review. BMJ Support Palliat Care. 2019;9(4):451-63.
11. Mody L, Miller DK, McGloin JM, Freeman M, Marcantonio ER, Magaziner J, et al. Recruitment and Retention of Older Adults in Aging Research. Journal of the American Geriatrics Society. 2008;56(12):2340-8.
12. Al Farsi RS, Al Alawi AM, Al Huraizi AR, Al-Saadi T, Al-Hamadani N, Al Zeedy K, et al. Delirium in Medically Hospitalized Patients: Prevalence, Recognition and Risk Factors: A Prospective Cohort Study. Journal of Clinical Medicine. 2023;12(12):3897.
13. Addesi D, Maio R, Smirne N, Laganà V, Altomari N, Puccio G, et al. Prevalence of Delirium in a Population of Elderly Outpatients with Dementia: A Retrospective Study. Journal of Alzheimer’s Disease. 2018;61(1):251-7.
